# Failure of standard methods for retrieving an unusual foreign body in esophagus: A case report

**DOI:** 10.1097/MD.0000000000018105

**Published:** 2019-11-27

**Authors:** Dongjie Li, Ling Nan, Kai Niu, Wanzhong Yin, Wei Zhu, Xin Wang

**Affiliations:** a Department of Otolaryngology-Head & Neck Surgery; b Department of Anesthesiology, First Hospital of Jilin University, Changchun 130021, Jilin Province, People's Republic of China.

**Keywords:** foreign body, esophagus, gallbladder grasping forceps

## Abstract

**Rationale:**

The ingestion of a foreign body (FB) with complete impaction of the esophagus is not common. Here we report a rare case of successful retrieval of a spherical stone in the esophagus of a man with mental retardation, using gallbladder grasping forceps and rigid endoscope.

**Patient concerns:**

A mental retarded man came to the emergency department presenting with recurrent nausea, vomiting, and dysphagia after swallowing a spherical stone. He had previously undergone an FB extraction under general anesthesia by fiberoptic esophagoscopy, which failed.

**Diagnosis:**

The diagnosis of FB ingestion was confirmed by anteroposterior plain film x-ray of the chest and chest computed tomography (CT), which showed the ingested spherical FB in the upper esophagus.

**Interventions:**

After multiple failed attempts using other instruments, the FB was successfully removed with gallbladder grasping forceps through a rigid esophagoscope.

**Outcomes:**

The patient was discharged without any complications. The nasogastric tube was extubated at the 10-day follow-up.

**Lessons subsections as per style:**

For esophageal retrieval of uncommon FBs, the instrument used is crucial. We report our experience retrieving a large and spherical FB in the upper esophagus using gallbladder grasping forceps. This proved to be an effective strategy, eliminating the need for thoracotomy.

## 1 Introduction

Foreign body (FB) ingestion is a relatively common emergency encountered in the field of otorhinolaryngology, it can be defined as materials swallowed accidentally or intentionally, or objects swallowed naturally when taking medication or food.^[1]^ Generally, the ingested FBs pass naturally and simply through the digestive tract without complication, however an estimated 10% to 20% of cases require endoscopic or surgical treatment.^[2,3]^ In adults, most FB ingestion occurs accidentally, but may be a result of contributory factors, such as psychiatric disorders, mental retardation, alcohol consumption, an edentulous state, or by those seeking secondary gain.^[4–6]^ FB impaction is varied and the risk to the patient ranges from negligible to life-threatening because of possible complications such as mucosal ulceration, esophageal perforation, mediastinitis, vascular trauma, aortoesophageal fistula, tracheoesophageal fistula, and others.^[7]^

The diagnosis and management of FBs mainly depend on the type and location of the foreign body. The first-tier treatment is endoscopic retrieval, but when endoscopic attempts fail and the clinical condition deteriorates, surgery is indispensable. However, an appropriate selection of instruments for uncommon FB removal is crucial during the whole procedure, and can shorten the operative duration and lessen potential complications. Here, we report a recurrent retrieval case of unusual FB impaction in the upper esophagus, which was finally removed using gallbladder-grasping forceps.

## 2 Case report

A 39-year-old man, with mental retardation and epileptic episodes since childhood, was transferred to the emergency department of our hospital in September, 2015. He was presented with a 24-hour history of recurrent nausea, vomiting, and dysphagia after ingesting a stone while playing outside the house, and was even unable to take water orally. His father reported that the patient had been admitted to a local hospital and underwent FB extraction under general anesthesia by fiberoptic esophagoscopy, which failed.

**Figure 1:**
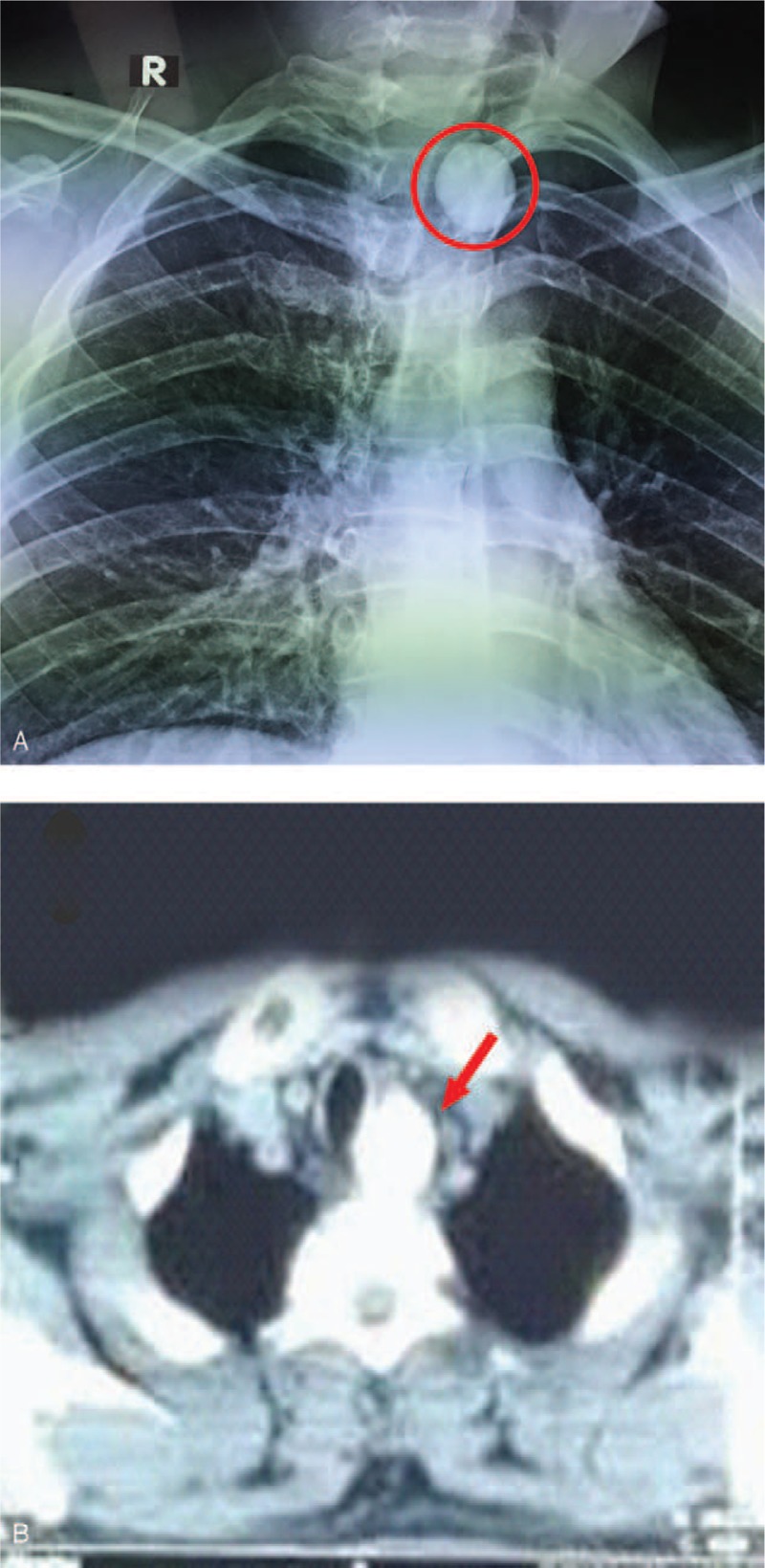
(A) Anteroposterior plain film X-ray. The red circle indicates the FB. (B) Chest CT scan. The red arrow indicates the FB.

At presentation, the patient appeared to be suffering and drooling. He was assessed by the otolaryngology team, owing to the history of FB ingestion. There was no cyanosis or loss of consciousness and the vital signs of the patient were normal. No other abnormalities were found on physical examination except tenderness above the upper sternal fossa. Anteroposterior plain film X-ray of the neck and chest computed tomography (CT) showed that the ingested nearly round FB was located in the upper esophagus (Fig. 1A). The FB measured almost 25 mm in diameter and 24 mm anteroposteriorly, based on the chest CT scan (Fig. 1B). No free air or pneumomediastinum was visible on the CT scan.

**Figure 2:**
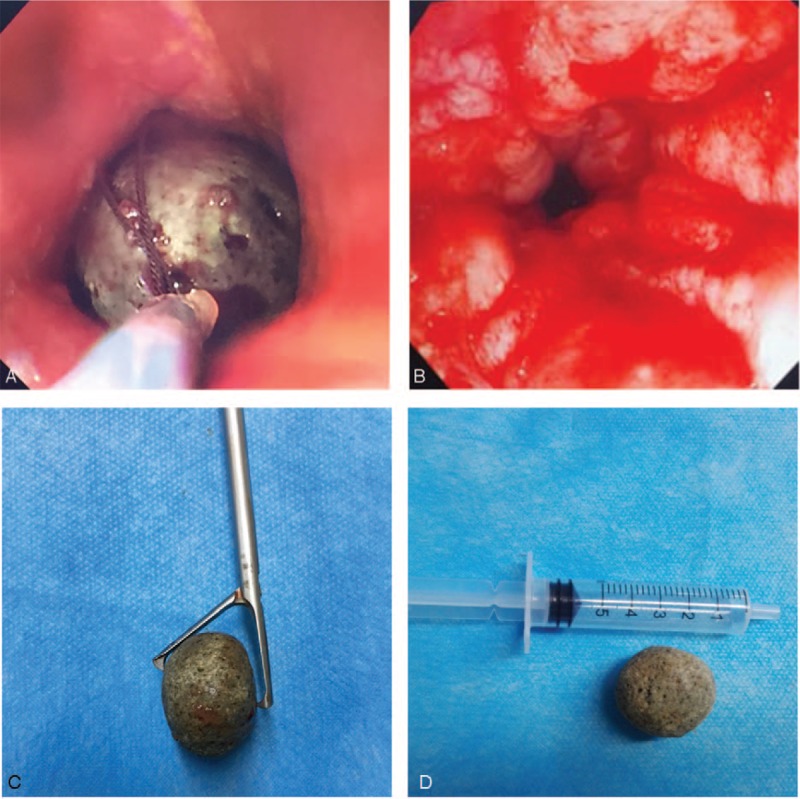
(A) Attempted retrieval of the FB via fiberoptic esophagoscopy with a snare. (B) Esophagus mucosal ulceration and granulation after FB removal. (C) Gallbladder-grasping forceps were able to hold the spherical FB tightly. (D) The diameter of the stone FB.

The man was shifted to the operating suite for emergency rigid esophagoscopy with the goal to extract the FB under general anesthesia. The smooth stone FB was found in the upper esophagus, 22 cm beyond the upper incisors. The surrounding mucosa was edematous and congested (Fig. 2A). The FB occupied the lumen and was impacted to the wall. All the various alligator forceps were tried in the FB extraction but failed, because the diameter of the FB (25 mm) was larger than the largest opening of the endoscopic forceps we had (20 mm). Subsequently, multiple attempts to retrieve the FB via fiberoptic esophagoscopy with a snare (Fig. 2A) and basket were also unsuccessful because of the hard texture of the FB stuck in the esophageal wall and its smooth surface.

Before we resorted to esophagectomy, finally gallbladder-grasping forceps were introduced through the rigid esophagoscope to successfully grasp the stone tightly and move it to the distal end of the rigid esophagoscope. The FB, forceps, and rigid esophagoscope were retrieved as a single unit. After retrieval of the FB, mucosal ulceration and granulation were noted around the site of impaction (Fig. 2B, 2C-2D), and a nasogastric tube was inserted. The patient was discharged uneventfully two days later.

**Figure 3:**
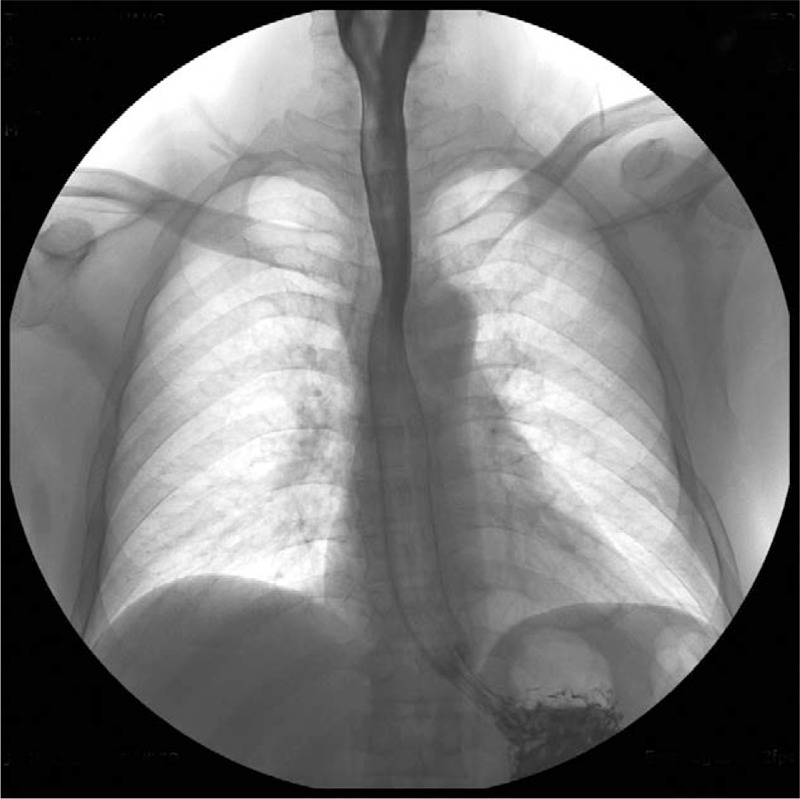
Postoperative radiological examination with Gastrografin.

The patient recovered well and a radiological examination with Gastrografin was performed at the 10-day follow-up (Fig. 3). X-rays showed no contrast medium spreading, and the antibiotic therapy and fasting were terminated.

## 3 Discussion

FB ingestion is one of the most common otorhinolaryngologic occurrences, often requiring urgent decision-making and management. In contrast to the high frequency of FB ingestion in children, occurrence in adults is relatively low, as 80% of FBs pass through the digestive system spontaneously without any intervention.^[8]^ The situation in adults occurs more commonly in those with psychiatric disorders, mental retardation, or impairment caused by alcohol.^[4,9–11]^

The types of FBs ingested vary by age and culture.^[1,3,5]^ In the current case, the patient was a 39-year-old male with mental retardation and episodes of epilepsy since childhood. The almost spherical stone (∼25 mm in diameter) was swallowed unintentionally. This is very rare and differed from previous reports in which the majority of FBs in adults were food, bones, or dental-related.^[12]^ The location of the FB in this case was in the upper esophagus, which is consistent with previous reports.^[3,7,13,14]^

After FB ingestion, patients were often present with clinical symptoms due to complete or incomplete esophageal obstruction. However, complete esophageal obstruction is not as common as incomplete. The clinical signs of complete esophageal obstruction are hypersalivation, drooling, inability to swallow liquids, and vomiting. Patients with these symptoms are at high risk for aspiration and require emergent endoscopic intervention.^[15]^

Some authors have recommended that endoscopic removal should be performed if esophageal FB impaction has lasted 24 hours, as the risk of major complications increases 14.1-fold afterward. Major complications include perforation with or without mediastinitis, retropharyngeal abscess, and aortoesophageal fistulae.^[16]^ The current case was rare, in that a middle-aged mentally retarded adult swallowed a ball-shaped stone that totally obstructed the esophagus. He was presented with repeated nausea, vomiting, and dysphagia, and tenderness above the upper sternal fossa. In this situation, urgent intervention was required to avoid serious complications.

The best method to remove an ingested esophageal FB is controversial. The main objective is to prevent further complications. Gastroenterologists advocate flexible instruments, but surgeons prefer rigid esophagoscopy. Both methods have been suggested because of their high detection, low complication, and high success rates.^[17]^

The choice of retrieval device is determined by the size and shape of the FB, the endoscope length and instrument channel, and by the endoscopist's preference and practice.^[2]^ Retrieval forceps have various types of jaw configurations: rat-tooth, alligator-tooth, or shark-tooth. Two- to five-prong forceps can be useful for retrieving soft objects, but not for harder or heavy objects because they cannot grasp securely enough. Polypectomy snares are widely available and inexpensive. Endoscopic baskets may be useful for some round objects, and retrieval nets or bags can provide a more secure grasp for some FBs, such as coins.^[2]^

However, the current case was challenged by a round stone with a smooth surface. In a local hospital, the patient underwent an attempted extraction by fiberoptic esophagoscopy under general anesthesia, and this failed. When we performed an emergency rigid esophagoscopy under general anesthesia after patient admission, the smooth stone totally occupied the lumen and pressed firmly and surrounded by the wall of the upper esophagus, together with surrounding edematous and congested mucosa. All the available traditional retrieval devices failed, for lack of purchase on this FB. Next, we tried a snare and basket retrieval under fiberoptic esophagoscopy, but that attempt also failed. In view of this situation, we used a gallbladder grasping forceps through the rigid esophagoscope and successfully extracted the stone. To our knowledge, the use of gallbladder grasping forceps was a novel application for FB ingestion retrieval.

According to our experience, the key factor in shortening procedural time is to catch and remove the FB in a short time using a powerful retrieval device. If the surface of the foreign body is smooth, it may be much more challenging for the operator. This is why it took us approximately 90 minutes to remove this FB. Fortunately, gallbladder grasping forceps were introduced, which has a large opening and more prongs to catch the FB and hold it securely, compared with traditional retrieval devices.

In conclusion, herein we reported our experience with retrieval of an ingested large and spherical FB from the upper esophagus, using gallbladder grasping forceps. This proved to be an effective strategy, and eliminated the need for thoracotomy.

## Author contributions

**Writing - Original Draft:** Dongjie Li.

**Resources:** Ling Nan.

**Investigation:** Kai Niu.

**Writing - Review & Editing:** Wanzhong Yin.

**Validation:** Wei Zhu.

**Supervision:** Xin Wang.

Dongjie Li orcid: 0000-0001-7560-0746.
